# Influence of LPCVD-Si_3_N_4_ Thickness on Polarization Coulomb Field Scattering in AlGaN/GaN Metal–Insulator–Semiconductor High-Electron-Mobility Transistors

**DOI:** 10.3390/mi16101147

**Published:** 2025-10-10

**Authors:** Guangyuan Jiang, Weikang Li, Xin Luo, Yang Liu, Chen Fu, Qingying Zhang, Guangyuan Zhang, Zhaojun Lin, Peng Cui

**Affiliations:** 1 School of Information Science and Electrical Engineering, Shandong Jiaotong University, Jinan 250357, China; 2 Shandong Key Laboratory of Technologies and Systems for Intelligent Construction Equipment, Shandong Jiaotong University, Jinan 250357, China; 3 Institute of Novel Semiconductors, Shandong University, Jinan 250100, China; 4 School of Integrated Circuits, Shandong University, Jinan 250101, China

**Keywords:** AlGaN/GaN MIS-HEMTs, gate dielectric layer, polarization Coulomb field scattering, two-dimensional electron gas, inverse piezoelectric effect

## Abstract

The thickness of the LPCVD-Si_3_N_4_ gate dielectric layer significantly influences the electron transport properties of AlGaN/GaN metal–insulator–semiconductor high-electron-mobility transistors (MIS-HEMTs), but the mechanism by which it affects polarization Coulomb field (PCF) scattering remains largely unexplored. In this study, AlGaN/GaN MIS-HEMTs with LPCVD-Si_3_N_4_ gate dielectric thicknesses of 0 nm, 5 nm, and 20 nm were fabricated, and the influence of LPCVD-Si_3_N_4_ thickness on PCF scattering was systematically investigated. Through electrical measurements and theoretical calculations, the relationship between LPCVD-Si_3_N_4_ gate dielectric layer thickness, additional polarization charge (∆*ρ*), two-dimensional electron gas (2DEG) density, and 2DEG mobility was analyzed. The results show that increasing the LPCVD-Si_3_N_4_ thickness reduces the vertical electric field in the AlGaN barrier, weakening the inverse piezoelectric effect (IPE) and reducing ∆*ρ*. Further analysis reveals that the ∆*ρ* exhibits a non-monotonic dependence on negative gate voltage, initially increasing and subsequently decreasing, due to the competition between strain accumulation and stress relaxation. Meanwhile, the 2DEG mobility limited by PCF (*μ*_PCF_) decreases monotonically with increasing negative gate voltage, mainly due to the progressive weakening of the 2DEG screening effect. The research results reveal the physical mechanism by which LPCVD-Si_3_N_4_ thickness regulates PCF scattering, providing theoretical guidance for optimizing gate dielectric parameters and enhancing the performance of AlGaN/GaN MIS-HEMTs.

## 1. Introduction

GaN is an outstanding representative of the new generation of semiconductor materials [1,2,3]. AlGaN/GaN HEMTs based on GaN materials have broad market application prospects in the power conversion field due to their excellent performance, including high electron mobility and high critical breakdown electric field [4,5].

In the field of power conversion, researchers typically insert a gate dielectric layer between the gate metal and barrier layer to fabricate MIS-HEMTs [6,7,8]. The insertion of a gate dielectric layer reduces gate leakage current [9,10]. Moreover, AlGaN/GaN MIS-HEMTs with a gate dielectric layer exhibit a larger gate swing, which facilitates higher current-driving capability and improved input-output voltage level matching in digital integrated circuits [11,12]. A variety of dielectric materials, such as Al_2_O_3_, SiO_2_, and Si_3_N_4_, have been employed as gate dielectrics [13,14]. Among them, low-pressure chemical vapor deposition (LPCVD)-Si_3_N_4_ has become an ideal choice for the gate dielectric layer due to its high dielectric constant, excellent thermal stability, and low defect density [13,15]. The thickness of the LPCVD-Si_3_N_4_ gate dielectric is a critical parameter influencing device characteristics. Previous studies have primarily focused on its effects on gate capacitance, threshold voltage, and gate leakage current [16,17,18]. However, little attention has been paid to the underlying mechanisms by which LPCVD-Si_3_N_4_ thickness influences barrier layer polarization and the scattering processes of channel electrons. In particular, the influence of LPCVD-Si_3_N_4_ gate dielectric thickness on polarization Coulomb field (PCF) scattering, which is related to barrier layer polarization and has a critical impact on the transport properties of the 2DEG [19,20,21], remains unclear. A complete 2DEG transport model can only be established by elucidating the correlation between the LPCVD-Si_3_N_4_ gate dielectric thickness and the various scattering mechanisms, which in turn provides theoretical guidance for optimizing the material parameters of the gate dielectric. Therefore, investigating the correlation between the LPCVD-Si_3_N_4_ gate dielectric thickness and scattering mechanisms such as PCF scattering is crucial for enhancing the performance of AlGaN/GaN MIS-HEMTs.

In this work, three AlGaN/GaN MIS-HEMT samples with LPCVD-Si_3_N_4_ gate dielectric thicknesses of 0 nm, 5 nm, and 20 nm were fabricated. Based on the measured electrical characteristics and theoretical calculations, the influence of LPCVD-Si_3_N_4_ thickness on PCF scattering and the underlying mechanism on device transport performance such as 2DEG mobility were systematically analyzed. The findings provide new theoretical insights into the role of gate dielectric thickness and offer guidance for optimizing dielectric parameters to enhance the performance of AlGaN/GaN MIS-HEMTs.

## 2. Experiments

In this study, the AlGaN/GaN wafer used for device fabrication was grown on SiC substrates by metal–organic chemical vapor deposition (MOCVD). The wafer structure consisted of a 100 nm AlN nucleation transition layer, a 1.5 μm GaN buffer, a 1 nm AlN interlayer, an 18 nm Al_0.2_Ga_0.8_N barrier, and a 2 nm GaN cap layer. During device fabrication, Si_3_N_4_ gate dielectrics with thicknesses of 5 nm and 20 nm were deposited by LPCVD. Mesa isolation was achieved by inductively coupled plasma (ICP) etching of LPCVD-Si_3_N_4_ and AlGaN/GaN layers with etching times of 3 min and 18 min, respectively. After etching, the mesa heights formed on the wafers with 5 nm and 20 nm LPCVD-Si_3_N_4_ were 136 nm and 150 nm, respectively. The source and drain electrodes were formed by ICP etching of LPCVD-Si_3_N_4_ for 4 min, followed by Ti/Al/Ni/Au deposition with thicknesses of 30/150/50/50 nm, respectively, by magnetron sputtering. A rapid thermal annealing process was then performed at 850 °C for 40 s to obtain ohmic contacts. Finally, Ni/Au with a thickness of 100/100 nm was deposited by magnetron sputtering to form the gate electrode.

**Figure 1:**
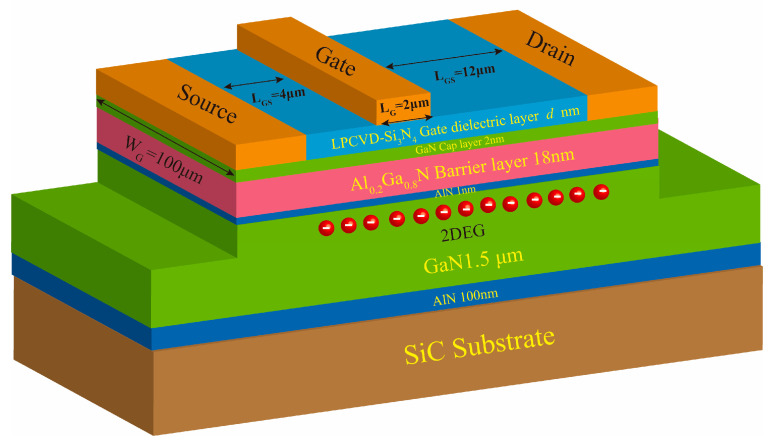
Schematic diagram of the device structure of the fabricated samples.

The AlGaN/GaN MIS-HEMTs fabricated with LPCVD-Si_3_N_4_ thicknesses of 5 nm and 20 nm were designated as Samples 2 and 3, respectively. In addition, an AlGaN/GaN HEMT with Schottky gate and without LPCVD-Si_3_N_4_ (0 nm) was fabricated and designated as Sample 1. The three samples were identical in terms of materials and structural parameters, except for the LPCVD-Si_3_N_4_ thickness. The three samples had a gate length (*L*_G_) of 2 μm, a gate width (W_G_) of 100 μm, a gate-source spacing (*L*_GS_) of 4 μm, and a gate–drain spacing (*L*_GD_) of 12 μm. The schematic diagram of the device structure of the fabricated samples is shown in Figure 1, where *d* (0 nm, 5 nm or 20 nm) indicates the LPCVD-Si_3_N_4_ thickness. The output current (I-V) and C-V characteristics of the samples were measured using a Keysight B1500 semiconductor device (Keysight Technologies; Santa Rosa; United States) parameter analyzer.

## 3. Results and Discussion

**Figure 2:**
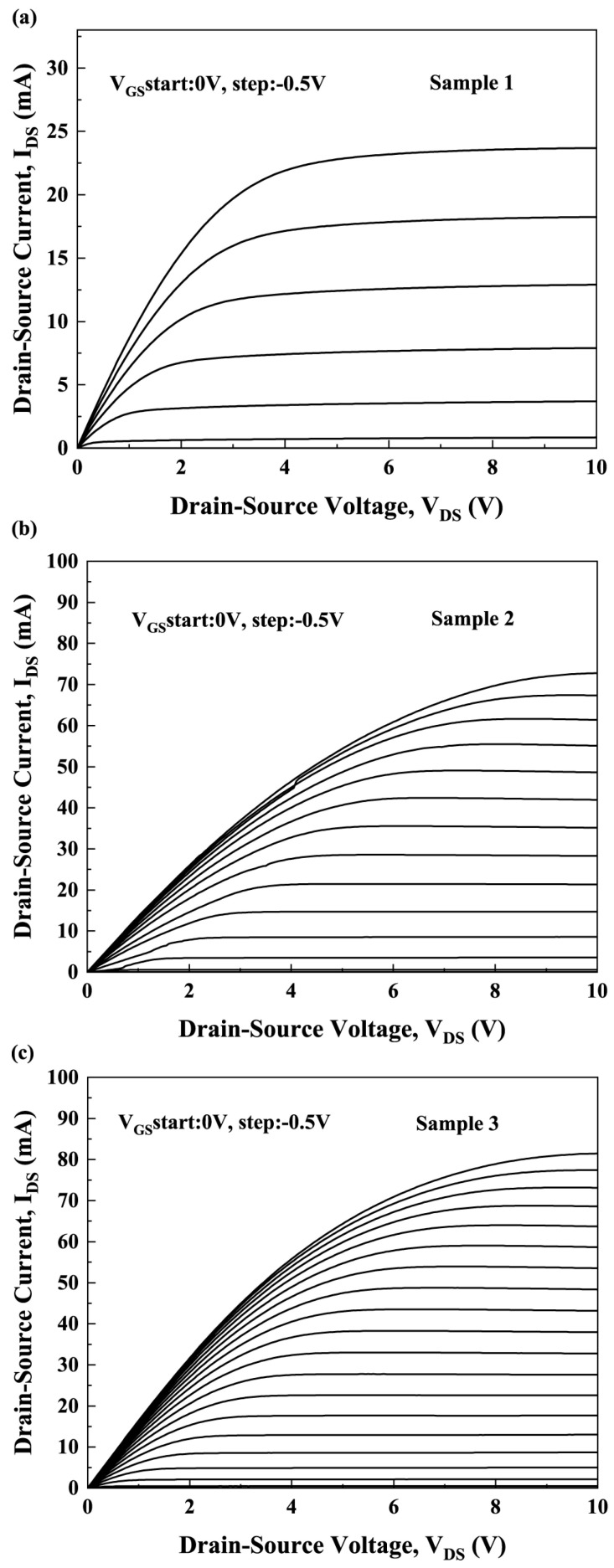
Measured I-V characteristics of (**a**) Sample 1, (**b**) Sample 2, and (**c**) Sample 3.

To investigate the influence of LPCVD-Si_3_N_4_ thickness on PCF scattering, the electron mobility limited by each scattering mechanism in the channel was calculated. For Sample 1, the electron mobilities limited by polar optical phonon (POP) scattering, piezoelectric (PE) scattering, acoustic deformation potential (DP) scattering, dislocation (DIS) scattering, and interface roughness (IFR) scattering were obtained [22]. For Samples 2 and 3, in addition to the above scattering mechanisms, the electron mobility limited by remote interface charge (RIC) scattering was also calculated, owing to the presence of fixed charges at LPCVD-Si_3_N_4_/GaN interface [23]. The electron mobility limited by RIC scattering can be calculated using the following relation [23,24]:

(1) $$\mu_{RIC}={\left[n_{fix}\cdot \frac{{\left(m^{*}\right)}^{2}}{2\pi \hbar^{3}k_{F}^{3}e}{\left(\frac{e^{2}}{2\varepsilon_{0}\varepsilon_{s}}\right)}^{2}\times \int_{0}^{2k_{F}}\frac{\exp^{-2qd_{fix}}}{{\left[q+q_{TF}G(q)\right]}^{2}}{\left(\frac{b}{b+q}\right)}^{6}\frac{q^{2}dq}{\sqrt{1-{\left(\frac{q}{2k_{F}}\right)}^{2}}}\right]}^{-1}$$

where $k_{F}={\left(2\pi n_{2DEG}\right)}^{\frac{1}{2}}$, $q_{TF}=\frac{m^{*}e^{2}}{2\pi \varepsilon_{0}\varepsilon_{s}\hbar^{2}}$ and $b={\left(\frac{33m^{*}e^{2}n_{2DEG}}{8\hbar^{2}\varepsilon_{0}\varepsilon_{s}}\right)}^{\frac{1}{3}}$. *n*_fix_ is the fixed charge at the LPCVD-Si_3_N_4_/GaN interface [24,25], the value of *n*_fix_ is calculated using the threshold voltage shift of a MIS-HEMT with an LPCVD-Si_3_N_4_ gate dielectric layer relative to a Schottky-gate HEMT. The threshold voltage shift can be expressed as [25]:

(2) $$V_{TH}^{MIS-HEMTs}-V_{TH}^{HEMTs}=\varphi_{b}^{i}-\varphi_{b}-\frac{1}{e}\Delta E_{C_{-}LPCVD-Si_{3}N_{4}/GaN}-V_{LPCVD-Si_{3}N_{4}}$$

where $\varphi_{b}^{i}$ is the barrier height of the Ni/LPCVD-Si_3_N_4_, $\varphi_{b}$ is the Schottky barrier height of the Ni/GaN, and $\Delta E_{C_{-}LPCVD-Si_{3}N_{4}/GaN}$ is the conduction band offset for LPCVD-Si_3_N_4_/GaN. $V_{LPCVD-Si_{3}N_{4}}$ can be expressed as [25]:

(3) $$V_{LPCVD-Si_{3}N_{4}}=\frac{e\left(n_{fix}-\sigma_{GaN}\right)}{C_{LPCVD-Si_{3}N_{4}}}$$

where $\sigma_{GaN}$ is net polarization charges at GaN surface, $C_{LPCVD-Si_{3}N_{4}}$ is capacitance of the LPCVD-Si_3_N_4_ gate dielectric layer. According to Equations (2) and (3), by substituting the known parameter values, the value of *n*_fix_ can be calculated [25]. Figure 2 shows the *I*-*V* characteristics of three samples at different gate voltages (*V*_GS_). The output current at a drain voltage (V_DS_) of 0.1 V was used to iteratively calculate the electron mobility and additional polarization charge (∆*ρ*) [22].

**Figure 3:**
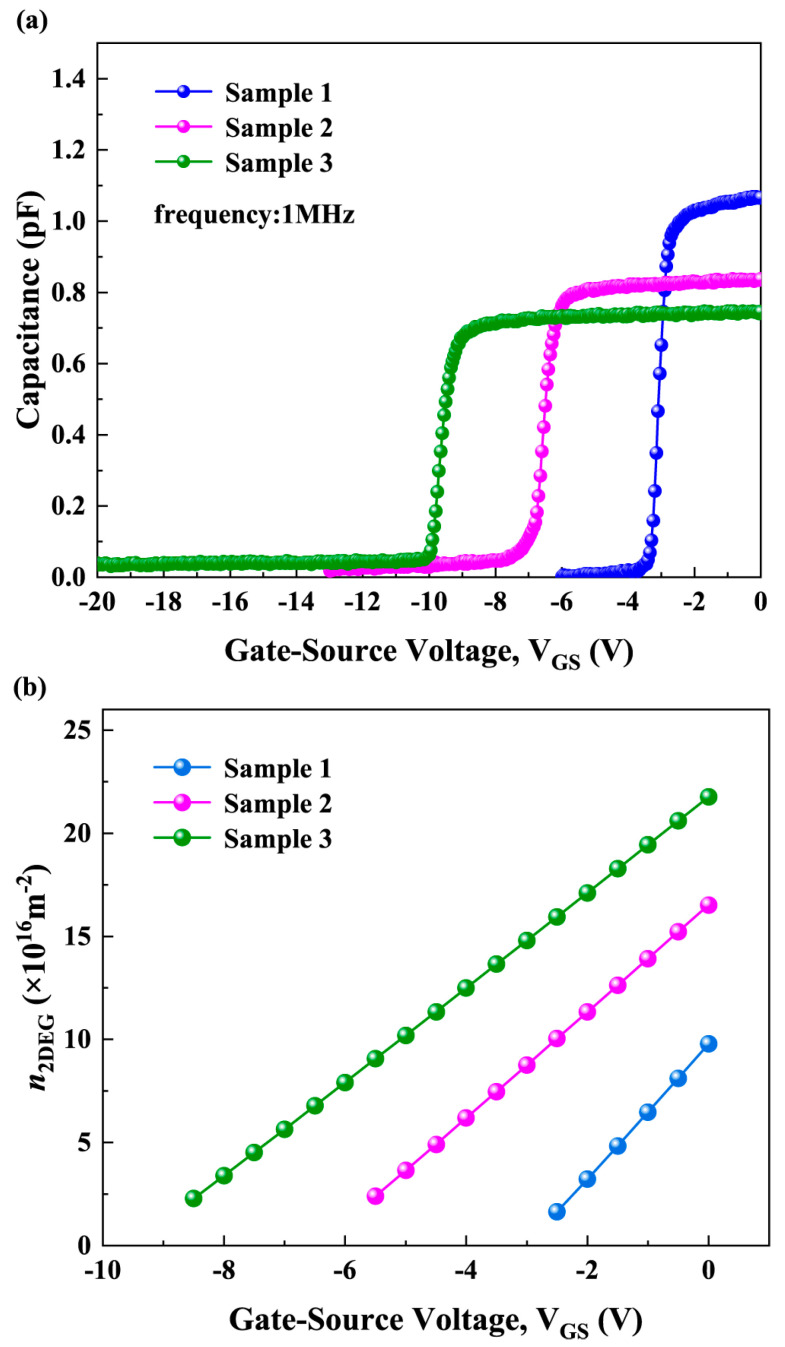
(**a**) Measured *C*-*V* curves and (**b**) calculated *n*_2DEG_ of three samples.

Figure 3a shows the measured *C*-*V* curve of three samples. The *n*_2DEG_ used in the calculations was derived from these curves. By differentiating the *C*-*V* curve in Figure 3a and identifying the peak of its derivative curve, the threshold voltage (*V*_Th_) of the sample was obtained [26]. The *n*_2DEG_ can then be calculated using equation [26,27,28]:

(4) $$n_{2\mathrm{DEG}}(V)=\frac{1}{S\cdot e}\int_{V_{\mathrm{Th}}}^{V}CdV$$

where *S* is the gate area and *e* is the elementary charge. The calculation results of *n*_2DEG_ are shown in Figure 3b. The total electron mobility is obtained based on Matthiessen rule using the electron mobility limited by each scattering mechanism. By calculating the momentum relaxation time for each scattering process and using the relationship between the momentum relaxation time, the effective mass of electrons in GaN, and the electron mobility, the electron mobility limited by each scattering mechanism can be determine. More details on the calculation process of electron mobility and ∆*ρ* can be found in reference [22].

**Figure 4:**
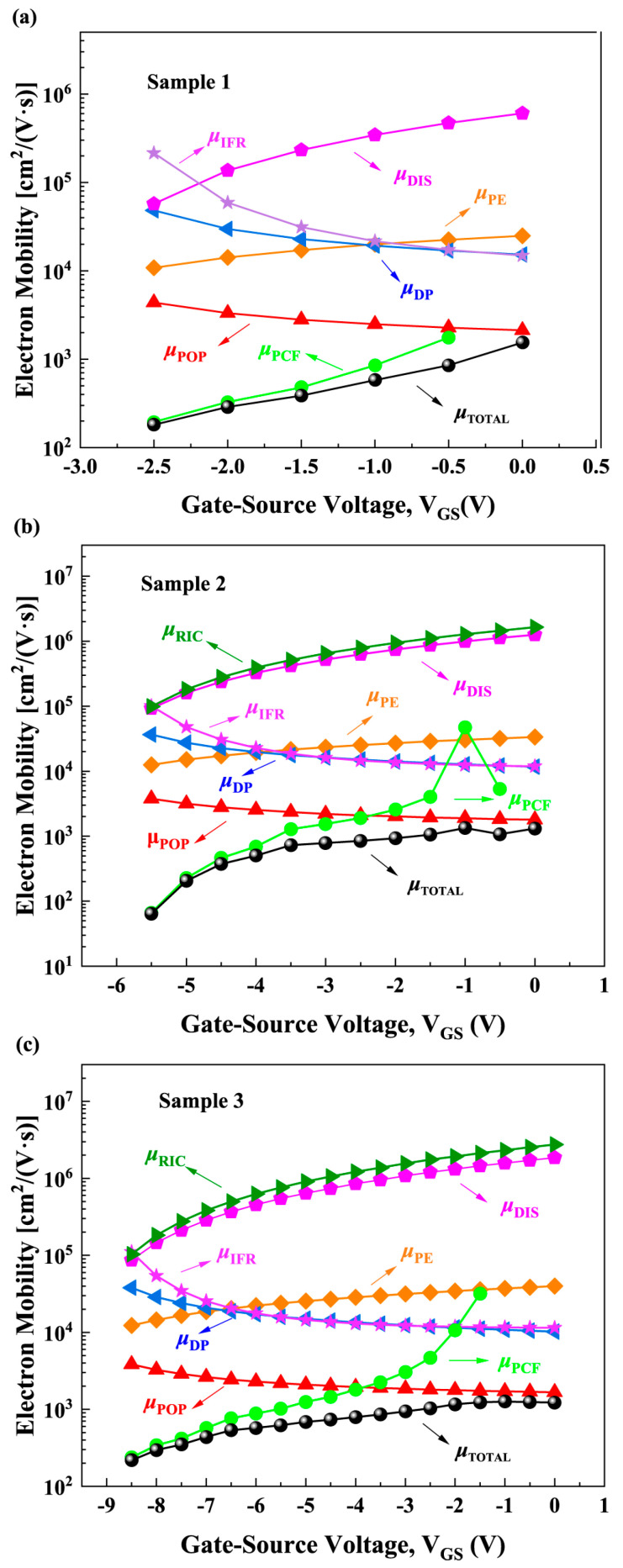
Calculated electron mobility for each scattering mechanism as a function of VGS for (**a**) Sample 1, (**b**) Sam-ple 2, and (**c**) Sample 3.

Figure 4 shows the calculated electron mobility limited by various scattering mechanisms. It can be seen that the *μ*_RIC_ value is large, which shows that it has little impact on *μ*_Total_ according to the Matthiessen rule [29]. This is because there is a GaN layer, an AlGaN barrier layer, and an AlN cap layer between the *n*_fix_ and the 2DEG, resulting in a relatively large separation distance of about 21 nm. Since RIC scattering is Coulomb interaction in nature, a larger distance leads to a weaker interaction, and thus the scattering effect of *n*_fix_ on the 2DEG is weakened. Although RIC scattering is unique to MIS-HEMTs with LPCVD-Si_3_N_4_ compared to Schottky gate HEMTs, its impact is small. Due to the influence of interface fixed positive charges and surface passivation effects, samples with different LPCVD-Si_3_N_4_ gate dielectric thicknesses exhibit different *n*_2DEG_ (as shown in Figure 3b). For POP, PE, DP, DIS, and IFR scattering, the LPCVD-Si_3_N_4_ gate dielectric thickness indirectly affects the electron mobilities limited by these scattering through its regulation of the channel *n*_2DEG_. As can be seen from Figure 4, the value of *μ*_PCF_ is small, which means that PCF scattering has a greater impact on *μ*_Total_ according to the Matthiessen rule. Therefore, it is crucial to clarify the influence of LPCVD-Si_3_N_4_ thickness on PCF scattering.

**Figure 5:**
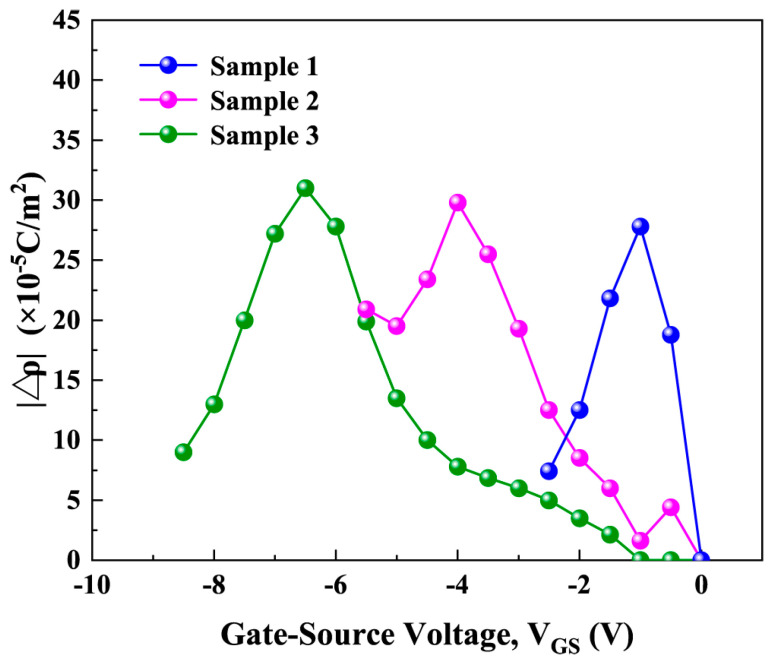
The calculated |∆*ρ*_G_| as a function of *V*_GS_.

**Figure 6:**
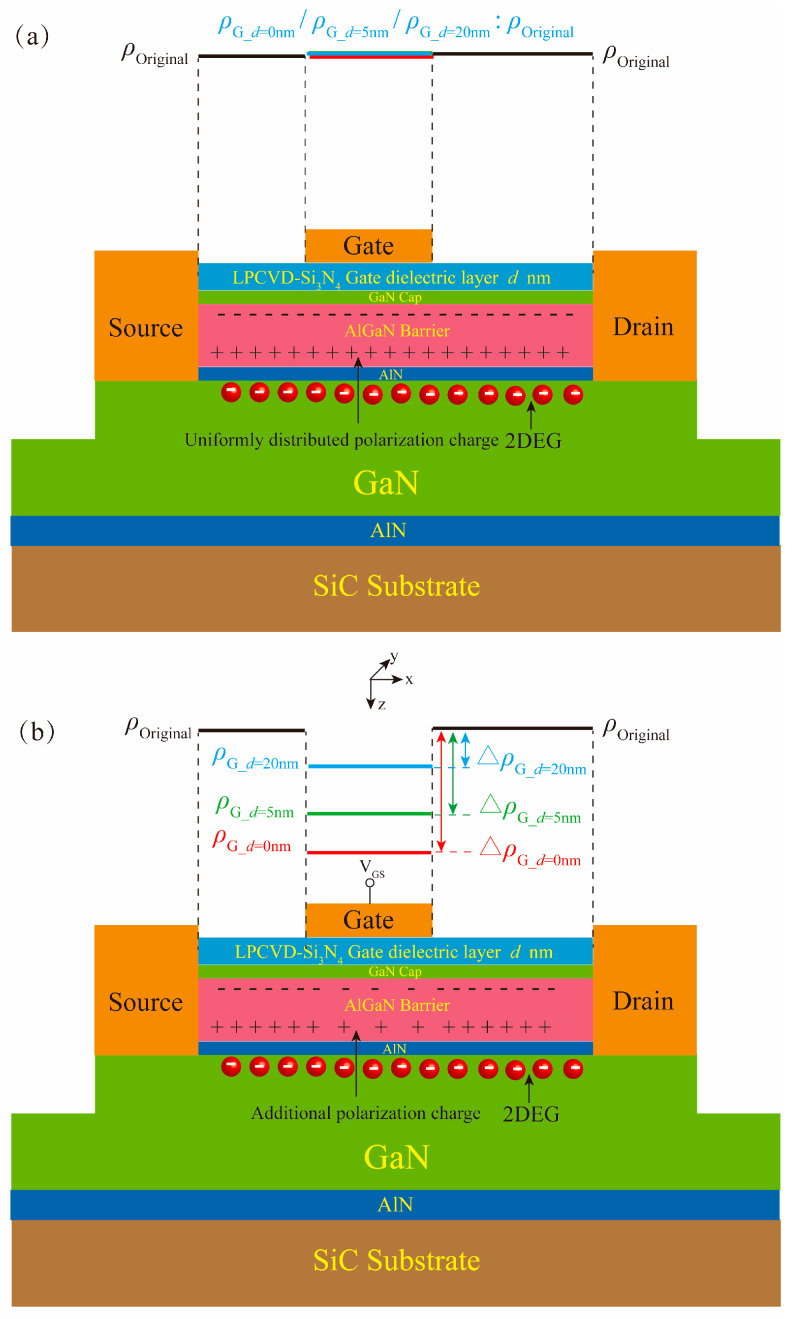
Schematic diagram of the polarization charge variation induced by *V*_GS_: (**a**) without *V*_GS_; (**b**) with *V*_GS_ applied.

PCF scattering is caused by the ∆*ρ*_G_ generated by the IPE under the gate [30]. Figure 5 shows the calculated absolute value of ∆*ρ*_G_ (|∆*ρ*_G_|) as a function of *V*_GS_. It can be seen from this Figure 5 that there are large differences in |∆*ρ*_G_| among the three samples. This is because the three samples have different LPCVD-Si_3_N_4_ thicknesses, resulting in different changes in polarization charge caused by *V*_GS_. As shown in Figure 6a, when *V*_GS_ is not added, the polarization charge under the gate of three samples is the *ρ*_Original_, which is the polarization charge of the material itself when no *V*_GS_ is applied (*ρ*_G_*d*=0nm/_*ρ*_G_*d*=5nm/_*ρ*_G_*d*=20nm_: *ρ*_Original_). To analyze the effect of different LPCVD-Si_3_N_4_ gate dielectric layer thickness on the electric field and polarization charge of the AlGaN barrier layer after applying *V*_GS_, a series capacitance model under the gate was established [31]. The gate-stack can be modeled as a series combination of the LPCVD-Si_3_N_4_ gate dielectric capacitor (*C*_LPCVD-Si3N4_) and the semiconductor capacitor (*C*_semi_) [31]. *C*_semi_ is the capacitance of the semiconductor layer consisting of the GaN cap layer, AlGaN barrier layer, AlN interlayer and 2DEG channel. When *V*_GS_ is applied, the total voltage is shared by these two capacitors, satisfying the following relationship:

(5) $$V_{GS}=V_{LPCVD-Si_{3}N_{4}}+V_{Semi}$$

where *V*_LPCVD-Si3N4_ is the voltage dropped on the LPCVD-Si_3_N_4_ gate dielectric layer and *V*_Semi_ is the effective voltage dropped across the semiconductor layers (including GaN cap layer, AlGaN barrier layer, AlN interlayer and 2DEG channel). According to the principle of capacitive voltage division, V_Semi_ can be given by the following formula:

(6) $$V_{Semi}=V_{GS}\cdot \frac{C_{LPCVD-Si_{3}N_{4}}}{C_{LPCVD-Si_{3}N_{4}}+C_{Semi}}$$

*C*_LPCVD-Si3N4_ follows the parallel plate capacitor formula, which is determined by its dielectric constant (ε), the dielectric constant of vacuum ($\varepsilon_{0}$), and thickness (*d*) [32]:

(7) $$C_{LPCVD-Si_{3}N_{4}}=\frac{\varepsilon \varepsilon_{0}}{d}$$

Substituting Formula (4) into Formula (3), we can obtain the relationship between V_Semi_ and *d*:

(8) $$V_{Semi}=V_{GS}\cdot \frac{1}{1+\frac{C_{Semi}}{\varepsilon \varepsilon_{0}}d}$$

When the same *V*_GS_ is applied, since the three samples have the same parameters except *d*, the larger *d* is, the smaller V_Semi_ is. The average vertical electric field in the AlGaN barrier layer ($E_{z}^{AlG\mathrm{aN}}$) is directly related to V_Semi_. Under low field conditions with a *V*_DS_ of 0.1 V, the channel potential is approximately 0, and the vertical electric field in the AlGaN can be expressed as:

(9) $$E_{z}^{AlG\mathrm{aN}}=\frac{V_{Semi}}{d_{eff}}$$

where *d*_eff_ is the effective thickness of semiconductor layer. Since the GaN cap layer and 2DEG layer are very thin, *d*_eff_ is approximately equal to the AlGaN thickness. It can be seen from Equations (8) and (9) that, under the same *V*_GS_, increasing the thickness of the LPCVD-Si_3_N_4_ gate dielectric layer can reduce the voltage drop on the semiconductor layer and effectively reduce the $E_{z}^{AlG\mathrm{aN}}$. Therefore, a thicker LPCVD-Si_3_N_4_ layer weakens the IPE in the AlGaN, resulting in a smaller ∆*ρ*. Figure 6b presents a schematic diagram of the ∆*ρ* distribution induced by *V*_GS_. These theoretical analyses are consistent with the calculated results shown in Figure 5. Under the same *V*_GS_ conditions, sample 3 with the thickest LPCVD-Si_3_N_4_ layer exhibits the smallest ∆*ρ*, while sample 1 without dielectric exhibits the largest ∆*ρ*.

As shown in Figure 5, the Δ*ρ* of the three samples exhibits a non-monotonic dependence on the *V*_GS_, initially increasing and subsequently decreasing. This behavior originates from the competition between strain accumulation induced by the IPE and strain relaxation associated with stress release. At the initial stage of applying a negative bias, the AlGaN barrier layer beneath the gate experiences a progressively increasing $E_{z}^{AlG\mathrm{aN}}$. This electric field induces a progressively increasing stress on the AlGaN lattice through the IPE. During this phase, the material remains within the elastic deformation regime, and the lattice strain increases linearly with the applied electric field. This strain directly modulates the piezoelectric polarization intensity, leading to an almost monotonic increase in the Δ*ρ* induced by the IPE with increasing negative gate bias. However, when the negative gate bias exceeds a critical value, the accumulated stress in the AlGaN layer surpasses its elastic limit. To accommodate this excessive stress, structural defects such as dislocations nucleate and propagate, effectively releasing the local strain [33]. Although the applied electric field continues to increase, the strain relaxation mechanism predominates, leading to a reduction in the effective piezoelectric polarization. Consequently, the Δ*ρ* no longer increases but decreases with further biasing.

**Figure 7:**
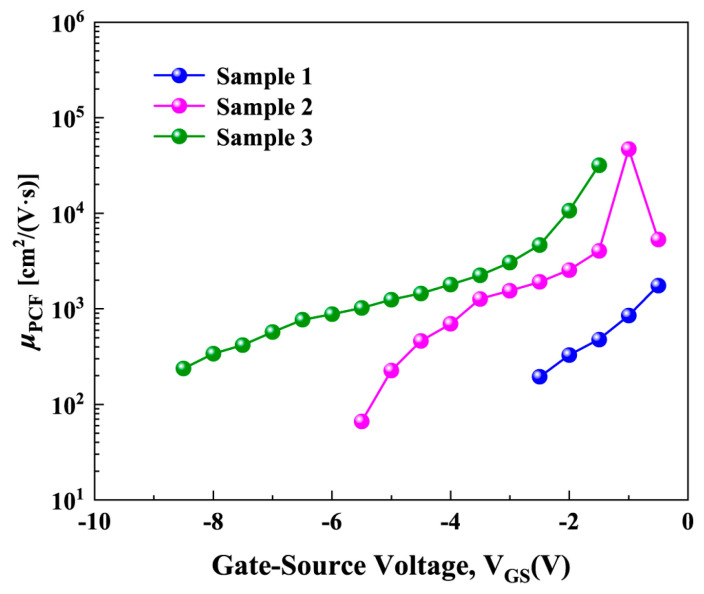
*μ*_PCF_ as a function of *V*_GS_ for the three samples.

Although Figure 5 shows that Δ*ρ* changes non-monotonically with increasing negative gate voltage, the *μ*_PCF_ in Figure 7 decreases monotonically with increasing negative gate voltage. This behavior can be attributed to the significantly weakened screening effect of the 2DEG. When calculating *μ*_PCF_, the screening effect of 2DEG is expressed by the screening function [34,35]:

(10) $$S(q,T_{e})=1+\frac{e^{2}F(q)\Pi (q,T_{e},E)}{2\varepsilon_{0}\varepsilon_{s}q}$$

where *T*_e_ is the electron temperature, $\Pi (q,T_{e},E)$ is the polarizability function. The form factor *F*(*q*) in the formula is [34,35]:

(11) $$F(q)=\int_{0}^{\infty }\int_{0}^{\infty }\psi^{2}\left(z\right)\psi^{2}(z^{\prime })\exp(-q\left|z-z^{\prime }\right|)dzdz^{\prime }$$

The wave function in the z direction is [34,35]:

(12) $$\Psi (z)={\left(b^{3}z^{2}/2\right)}^{1/2}\exp(-bz/2)$$

In order to minimize the energy of the 2DEG system, the variational parameter is [34,35]:

(13) $$b={\left(33m^{*}e^{2}n_{2\mathrm{DEG}}/8\varepsilon_{s}\hbar^{2}\right)}^{1/3}$$

Therefore, based on the above Equations (10)–(13), the screening effect is closely related to *n*_2DEG_. With increasing negative gate voltage, *n*_2DEG_ decreases monotonically (as shown in Figure 3b), leading to a reduction in its screening ability. Even when Δ*ρ* begins to decrease after reaching a peak under large negative gate voltages, the reduced screening ability continues to enhance the influence of the scattering potential corresponding to Δ*ρ* on electrons, thereby causing *μ*_PCF_ to decrease monotonically. These results indicate that *μ*_PCF_ depends not only on the magnitude of Δ*ρ* but is also strongly modulated by the 2DEG screening effect.

## 4. Conclusions

In summary, this work systematically investigates the influence of LPCVD-Si_3_N_4_ gate dielectric layer thickness on PCF scattering and the transport properties of AlGaN/GaN MIS-HEMTs. The results demonstrate that increasing the LPCVD-Si_3_N_4_ gate dielectric thickness effectively reduces the $E_{z}^{AlG\mathrm{aN}}$, thereby weakening the IPE and reducing the generation of ∆*ρ*. The ∆*ρ* exhibits a non-monotonic dependence on negative gate bias, initially increasing and then decreasing due to IPE and the competition between strain accumulation and stress relaxation. However, the *μ*_PCF_ decreases monotonically with negative gate bias, primarily due to the weakening of the 2DEG screening effect. Comprehensive analysis indicates that the variation in *μ*_PCF_ in MIS-HEMTs is influenced not only by the polarization charge modulated by the LPCVD-Si_3_N_4_ thickness, but also closely related to the screening ability of 2DEG. This study reveals the mechanism by which gate dielectric thickness modulates PCF scattering, providing a theoretical basis and design reference for optimizing gate dielectric parameters and improving the electron transport performance of AlGaN/GaN MIS-HEMTs. In future work, the relationship between 2DEG mobility and gate bias could be utilized to study the subthreshold swing. Furthermore, by measuring the device’s S-parameters, a small-signal equivalent circuit model can be developed to analyze the effects of PCF scattering on 2DEG mobility, access resistance and transconductance, and explore approaches for improving device linearity.

## Data Availability

The original contributions presented in this study are included in the article. Further inquiries can be directed to the corresponding author.
